# Complete plastome sequence of *Hydnocarpus hainanensis* Merr (Achariaceae): an endemic ‘vulnerable’ tree in South China

**DOI:** 10.1080/23802359.2018.1511853

**Published:** 2018-10-08

**Authors:** Chao-Rui Chen, Jian-Hua Wang, Kun-Kun Zhao, Zhi-Xin Zhu, Hua-Feng Wang

**Affiliations:** Hainan Key Laboratory for Sustainable Utilization of Tropical Bioresources, Institute of Tropical Agriculture and Forestry, Hainan University, Haikou, China

**Keywords:** Hydnocarpus hainanensis Merr., plastome, phylogeny, genome structure, Achariaceae

## Abstract

*Hydnocarpus hainanensis* Merr. is an evergreen tree with a height of 6–12 m and a diameter at breast height of 50 cm. It is distributed in Guangxi, Guizhou, Hainan, South of Yunnan Province of China. Here, we report and characterize the complete plastid genome sequence of *H. hainanensis* in an effort to provide genomic resources useful for promoting its conservation and systematics research. The plastome of *H. hainanensis* is found to possess a total length 163,330 bp with the typical quadripartite structure of angiosperms, containing two inverted repeats (IRs) of 26,870 bp, a large single copy (LSC) region of 91,510 bp and a small single copy (SSC) region of 18,080 bp. The plastome contains 111 genes, consisting of 78 unique protein-coding genes (seven of which are duplicated in the IR: *rps12*, rps*7*, ndh*B*, ycf*2*, rpl*23*, rpl*2*, and *rps19*), 29 unique tRNA genes (seven of which are duplicated in the IR, i.e. *trnN^GUU^*, *trnR^ACG^, trnA^UGC^, trnl^GAU^, trnV^GAC^, trnL^CAA^*, and *trnl^CAU^*) and four unique rRNA genes (5S rRNA, 4.55S rRNA, 23S rRNA, and 16S rRNA). The overall A/T content in the plastome of *H. hainanensis* is 63.70%. The phylogenetic analysis indicated that *H. hainanensis* is close to *Salix rorida* within Malpighiales. The complete plastome sequence of *H. hainanensis* will provide a useful resource for the conservation genetics of the one species as well as for the phylogenetic studies of Achariaceae.

## Introduction

*Hydnocarpus hainanensis* is an evergreen tree with a height of 6–12 m and a diameter at breast height of 50 cm. It is distributed throughout the evergreen broad-leaved forests having altitudes that range from 300 to 1800 m in Guangxi, Guizhou, Hainan, South of Yunnan Province in China (Yang and Sue 2007). It has been ranked as a VU (vulnerable) species in China (Ministry of Environmental Protection of the People’s Republic of China and Chinese Academy of Sciences 2013). In China, this species is under threat because of its habitat loss and harvesting of the durable and decay-resistant timber and the fruit for the treatment of skin conditions. Besides, its natural regeneration is poor. Consequently, its genetic and genomic information are urgently needed in order to promote its conservation and economic use of *H. hainanensis*. Here, we report and characterize the complete plastome of *H. hainanensis* (GenBank accession number: MH708163, this study). This is the first report of a complete plastome for the *H. hainanensis*.

In this study, *H. hainanensis* is sampled from Diaoluo Mountain (18.67°N, 109.88°E), which is a National Nature Reserve of Hainan, China. The voucher specimens (Wang et al. B50) are deposited in the Herbarium of the Institute of Tropical Agriculture and Forestry (HUTB), Hainan University, Haikou, China.

The modified CTAB method of Doyle and Doyle (1987) is used to extract total genomic DNA from leaves quickly frozen with dry ice. One microgram of genomic DNA is used for Illumina library preparation, using version 3 chemistry. Paired-end, 150 bp reads are sequenced using an Illumina HiSeq 2500 platform at the Guangzhou Novel-seq Biotechnology Co., Ltd. (Guangzhou, China). Reads are trimmed and those with >10% Ns or with >10% low-quality (*Q* ≤ 5) bases are filtered out using NGSQC-Toolkit v2.3.3 (Patel and Jain 2012). Cleaned reads are assembled against the plastome of *Idseia polycarpa* (NC_032060.1) (Yang et al. 2016) using MITObim v1.8 (Hahn et al. 2013). The plastome is annotated using Geneious R8.0.2 (Biomatters Ltd., Auckland, New Zealand) against the plastome of *Idseia polycarpa* (NC_032060.1). The annotation is corrected with DOGMA (Wyman et al. 2004).

The plastome of *H. hainanensis* is found to possess a total length 163,330 bp with the typical quadripartite structure of angiosperms, containing two inverted repeats (IRs) of 26,870 bp, a large single copy (LSC) region of 91,510 bp and a small single copy (SSC) region of 18,080 bp. The plastome contains 111 genes, consisting of 78 unique protein-coding genes (seven of which are duplicated in the IR, i.e. *rps12*, rps*7*, ndh*B*, ycf*2*, rpl*23*, rpl*2*, and *rps19*), 29 unique tRNA genes (seven of which are duplicated in the IR, i.e. *trnN*-^GUU^, trn*R*-^ACG^, trn*A*-^UGC^, trn*l*-^GAU^, trn*V*-^GAC^, trn*L*-^CAA^, and *trnl*-^CAU^) and four unique rRNA genes (5SrRNA, 4.55SrRNA, 23S rRNA, and 16SrRNA). Among these gene, one pseudogene (*ndhF*, translation from 120,829 to 118,625), 13 genes (*trnV*-^UAC^, trn*L*-^UAA^, trn*I*-^GAU^, trn*K*-^UUU^, trn*A*-^UGC^, atp*F*, pet*B*, pet*D*, rpo*C1*, rpl*16*, rpl*2*, ndh*B*, and *ndhA*) possessed a single intron and three genes (*ycf3*, clp*P*, and *rps12*) had two introns. The gene *rps12* is found to be trans-spliced, as is typical of angiosperms. The overall A/T content in the plastome of *H. hainanensis* is 63.70%, in which the corresponding value of the LSC, SSC, and IR region is 66.10%, 69.50%, and 57.50%, respectively.

**Figure 1.**
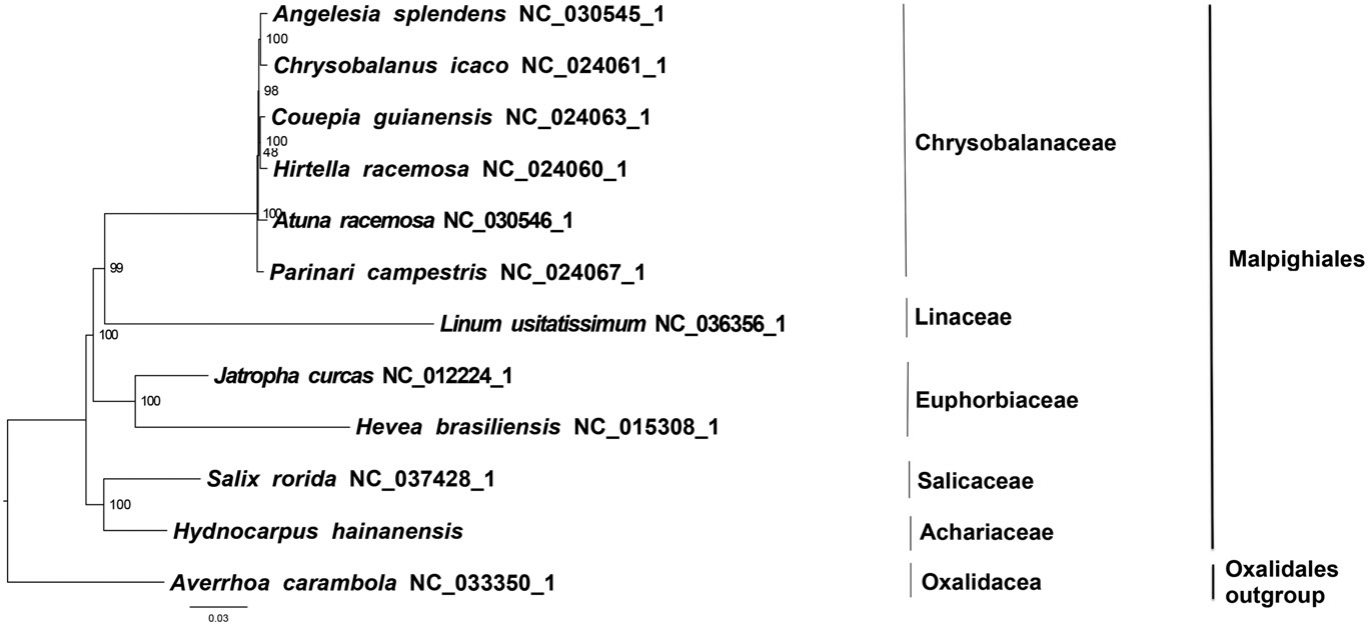
The best ML phylogeny recovered from 12 complete plastome sequences by RAxML. Accession numbers: *Hydnocarpus hainanensis* (MH708163, this study)*, Linum usitatissimum *NC_036356.1*, Salix rorida *NC_037428.1*, Chrysobalanus icaco *NC_024061.1*, Parinari campestris *NC_024067.1*, Couepia guianensis *NC_024063.1*, Hirtella racemosa *NC_024060.1*, Hevea brasiliensis *NC_015308.1*, Jatropha curcas *NC_012224.1*, Atuna racemosa *NC_030546.1*, Angelesia splendens *NC_030545.1*;* outgroup: *Averrhoa carambola *NC_033350.1*.*

We used RAxML (Stamatakis 2006) with 1000 bootstraps under the GTRGAMMAI substitution model to reconstruct a maximum likelihood (ML) phylogeny of 10 published complete plastome of Malpighiales, using *Averrhoa carambola* (Oxalidales, Oxalidaceae) as outgroup. The phylogenetic analysis indicated that *H. hainanensis* is closer to *Salix rorida* than other taxa within Malpighiales (Figure 1). Most nodes in the plastome ML trees are strongly supported. The complete plastome sequence of *H. hainanensis* will provide a useful resource for the conservation genetics of the species as well as for the phylogenetic studies of Achariaceae.
